# Herpes simplex virus 1 inhibits phosphorylation of RNA polymerase II CTD serine-7

**DOI:** 10.1128/jvi.01178-24

**Published:** 2024-09-24

**Authors:** Adam W. Whisnant, Oliver Dyck Dionisi, Valeria Salazar Sanchez, Julia M. Rappold, Lara Djakovic, Arnhild Grothey, Ana Luiza Marante, Patrick Fischer, Shitao Peng, Katharina Wolf, Thomas Hennig, Lars Dölken

**Affiliations:** 1 Institute for Virology and Immunobiology, Julius-Maximilians-University Würzburg, Würzburg, Germany; 2 Hannover Medical School, Institute of Virology, Hannover, Germany; University of Virginia, Charlottesville, Virginia, USA

**Keywords:** herpes simplex virus, RNA polymerases, transcription

## Abstract

Transcriptional activity of RNA polymerase II (Pol II) is influenced by post-translational modifications of the C-terminal domain (CTD) of the largest Pol II subunit, RPB1. Herpes simplex virus type 1 (HSV-1) usurps the cellular transcriptional machinery during lytic infection to efficiently express viral mRNA and shut down host gene expression. The viral immediate-early protein ICP22 interferes with serine 2 phosphorylation (pS2) by targeting CDK9 and other CDKs, but the full functional implications of this are not well understood. Using Western blotting, we report that HSV-1 also induces a loss of serine 7 phosphorylation (pS7) of the CTD during lytic infection, requiring expression of the two immediate-early proteins ICP22 and ICP27. ICP27 has also been proposed to target RPB1 for degradation, but we show that pS2/S7 loss precedes the drop in total protein levels. Cells with the RPB1 polyubiquitination site mutation K1268R, preventing proteasomal degradation during transcription-coupled DNA repair, displayed loss of pS2/S7 but retained higher overall RPB1 protein levels later in infection, indicating this pathway is not involved in early CTD dysregulation but may mediate bulk protein loss later. Using α-amanitin-resistant CTD mutants, we observed differential requirements for Ser2 and Ser7 for the production of viral proteins, with Ser2 facilitating viral immediate-early genes and Ser7 appearing dispensable. Despite dysregulation of CTD phosphorylation and different requirements for Ser2/7, all CTD modifications tested could be visualized in viral replication compartments with immunofluorescence. These data expand the known means that HSV employs to create pro-viral transcriptional environments at the expense of host responses.

**IMPORTANCE:**

Cells rapidly induce changes in the transcription of RNA in response to stress and pathogens. Herpes simplex virus (HSV) disrupts many processes of host mRNA transcription, and it is necessary to separate the actions of viral proteins from cellular responses. Here, we demonstrate that viral proteins inhibit two key phosphorylation patterns on the C-terminal domain (CTD) of cellular RNA polymerase II and that this is separate from the degradation of polymerases later in infection. Furthermore, we show that viral genes do not require the full “CTD code.” Together, these data distinguish multiple steps in the remodeling of RNA polymerase during infection and suggest that shared transcriptional phenotypes during stress responses do not revolve around a core disruption of CTD modifications.

## INTRODUCTION

Herpes simplex virus type 1 (HSV-1) is a large, double-stranded DNA virus present in nearly two-thirds of the global population that is the causative agent of the common cold-sore as well as severe skin lesions, life-threatening neonatal encephalitis, and a leading cause of infectious blindness (1). HSV-1 is a paradigm for a virus, which induces a profound host shut-off during productive infection by targeting multiple steps of RNA metabolism.

RNA polymerase (Pol) II is the complex responsible for transcription of all mRNA and several non-coding RNAs. Pol II is a multi-subunit complex with the most dynamic regulatory events occurring on the C-terminal domain (CTD) of the largest subunit, RPB1. The RPB1 CTD consists of heptapeptide repeats of the evolutionarily conserved consensus sequence tyrosine-serine-proline-threonine-serine-proline-serine (Y1-S2-P3-T4-S5-P6-S7) (2). Several non-consensus heptapeptide repeats, particularly with variations in the seventh amino acid position, are enriched in the more distal of the 52 mammalian CTD repeats. Each consensus non-proline residue serves as a site of phosphorylation that helps coordinate the different steps in transcription, with numerous additional post-translational modifications having been described in both the CTD and other regions of RPB1 (3).

Within hours of entering a cell, HSV is able to globally interfere with multiple Pol II processes on host genes such as promoter clearance (4, 5), promoter-proximal pausing (6, 7), and polyadenylation (8) while preserving these functions on viral genes. This selective permissiveness has been attributed to different mechanisms, including bulk redirection of DNA-binding proteins to nucleosome-free viral DNA (5, 9) to the regulation of specific activities such as mRNA 3’-end formation by the Cleavage and Polyadenylation Specificity Factor (CPSF). Via its interaction with CPSF, the HSV-1 immediate early protein ICP27 induces the assembly of a dead-end 3’ processing complex, blocking mRNA cleavage. However, 3’-end processing of viral (and a subset of host) transcripts is rescued by the RNA sequence-dependent binding/activator activity of the viral ICP27 protein (10). The viral protein ICP22 has also been shown to interact with Cyclin-dependent kinase 9 (CDK9) (11, 12) and lead to a decrease of phosphorylated CTD Ser2, regarded as a positive marker for transcriptional elongation and processivity (13). ICP22 was recently shown to interact with other Ser2 kinases, such as CDKs 11, 12, and 13 (14).

Pol II activity is also tightly regulated under conditions of abiotic stress. For instance, a failure of polyadenylation has been shown under conditions of hypoxia (15); heat, osmotic, and oxidative stress (16–19); as well as in renal carcinoma (20). Under these conditions, the normal 3’ ends of mRNA are not formed, and polymerases transcribe thousands of additional bases downstream of normal termination sites, termed disruption of transcription termination (DoTT), downstream of gene (DoG) transcription, or read-through of polyadenylation (polyA) sites/polyA read-through. Distinguishing which processes are directly regulated by viral proteins and which are disrupted by cellular stress responses becomes imperative to understand transcription during infection.

In this study, we sought to determine HSV-1-induced dysregulation of Pol II CTD modifications and their functional consequences in relation to other stressors. Besides the well-described loss of Ser2 phosphorylation (pS2), HSV-1 infection of non-transformed human fibroblasts also resulted in a global loss of Ser7 phosphorylation (pS7) by 8 h post-infection (p.i.), which was not observed in cellular stress responses and is necessary for the termination of Pol II-derived snRNA. Expression of the two viral immediate-early genes ICP22 and ICP27 was necessary to induce the loss of pS7, and we provide additional evidence that loss of CTD hyperphosphorylation is separate from bulk RPB1 degradation. Although phosphorylation of both residues is reduced in infection, alanine substitution of Ser7 had no major impact on viral gene expression while Ser2 substitution was detrimental. Despite Ser7 being dispensable for viral gene expression, its phosphorylation, as well as every other CTD modification examined, could be visualized in viral replication compartments in non-transformed cells. These findings expand the known means of transcriptional regulation by a major human pathogen.

## RESULTS

### Quantification of CTD modifications during conditions of polyA-site read-through

**Fig 1:**
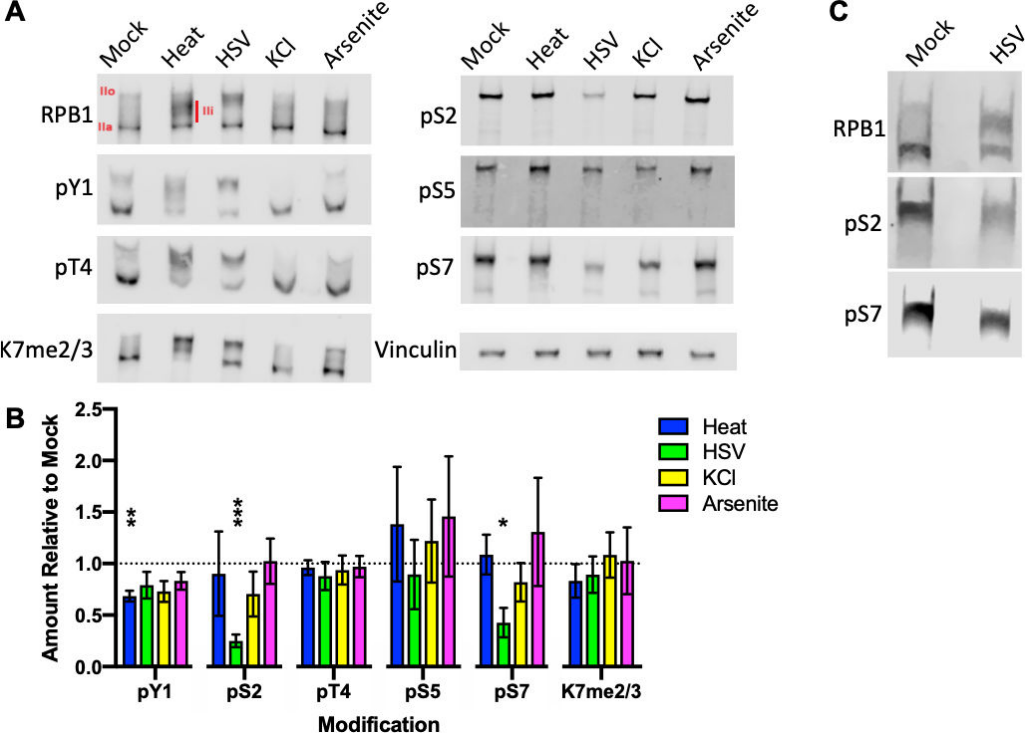
Western blot quantification of RPB1 CTD modifications during conditions of polyA-site read-through. (**A**) Human foreskin fibroblasts were subjected to heat (44°C, 2h), HSV infection (strain 17syn+, MOI 10, 8h), osmotic (80 mM KCl, 1h), and oxidative (0.5mM NaAsO_2_, 1 h) stress, and total protein was harvested at the end of the stress period. Samples were resolved by SDS-PAGE and probed for levels of RPB1, its CTD modifications, and vinculin as a housekeeping control. The major migrating forms of RPB1 (IIo, IIi, and IIa) are indicated in the RPB1 blot. (**B**) Signals for all forms of each CTD modification were measured on an Odyssey Fc fluorimeter, normalized to the signal for all forms of RPB1 on the same blot, and plotted relative to levels in untreated cells. Mean values of three biological replicates with standard deviations are plotted. Statistically significant differences to mock are indicated as *P < 0.05, **P < 0.01, ***P < 0.001. (**C**) Western blot samples of RPB1 and phosphoserine levels in infected differentiated neurons (strain 17syn+, MOI 10, 8 h), representative of three biological replicates.

Considerable overlap exists between genes exhibiting defective polyadenylation during different conditions of osmotic or heat stress and herpes simplex virus infection (21). As transcriptional termination is coordinated in part by CTD phosphorylation, we first determined whether a common disruption of RPB1 modifications exists under conditions of heat stress (44°C, 2 hours [h]), HSV infection (strain 17syn+, MOI = 10, 8 h), osmotic stress (80 mM KCl, 1 h), or oxidative stress induced by 0.5 mM sodium arsenite for 1 h in human fibroblasts. Time points for each condition were based on use in studies cited above. RPB1 migration in sodium dodecyl sulfate (SDS) gels can be altered by the degree of post-translational modification, both by slight increases in molecular weight but primarily by the negative charges of phosphates at gel pH altering stoichiometric association with negatively charged detergents. RPB1 migrates closer to its native molecular weight when unphosphorylated, dubbed Pol IIa, whereas hyperphosphorylated RPB1 exhibits substantially slower migration and travels as form IIo. Both heat stress and HSV infection induced accumulation of an intermediately phosphorylated RPB1 band (IIi) as seen by relative migration in SDS-PAGE (Fig. 1A).

Given the various migration patterns across all samples, we thus quantified signals for CTD modifications across the entire range of RPB1 bands and normalized them to total RPB1 signals on the same blot. For all stress conditions, a slight (20%–30% on average) reduction in tyrosine 1 phosphorylation (pY1) was observed relative to mock (Fig. 1B). Dephosphorylation of Y1 is directly involved in recruiting termination factors in yeast (22), and mammalian RPB1 mutants where the distal 3/4 of Y1’s are substituted for phenylalanine also exhibit termination defects (23), although it is unclear if a ~20% global reduction in pY1 would be sufficient to recapitulate this phenotype. Surprisingly, threonine 4 phosphorylation (pT4), which predominately accumulates around transcription termination sites and is involved in 3’-end formation (24, 25), remained unaffected in all conditions; as did serine 5 phosphorylation (pS5) and K7 di/tri-methylation levels (K7me). HSV infection was the only condition observed to heavily impact CTD modifications, causing a ~70% reduction in pS2, as previously described, and a ~50% loss of serine 7 phosphorylation (pS7). The reduction in both pS2/7 by 8 h could also be observed in post-mitotic neurons differentiated from Lund human mesencephalic (LUHMES) cells (Fig. 1C), which have recently been established as a model for HSV latency (26).

As previous studies have shown binding of monoclonal antibodies to the CTD can be affected by the identity and phosphorylation of nearby amino acids (27–30), we tested a panel of CTD phospho-serine antibodies on the same samples. The data for CTD phospho-serines in Fig. 1 are for selected antibodies whose epitope biases have been evaluated by in other studies (27, 29); nevertheless, we tested additional phospho-serine antibodies. The results for HSV infection were consistent with all CTD antibodies tested (a total of five, three, and two antibodies for pS2, pS5, and pS7, respectively, Fig. S1). Overall, these data indicate that there is not a major CTD modification indicative of general stress responses and that HSV-1 causes a loss of two CTD modifications in multiple cell types.

### Loss of CTD serine 7 phosphorylation in HSV infection requires expression of both ICP22 and ICP27

**Fig 2:**
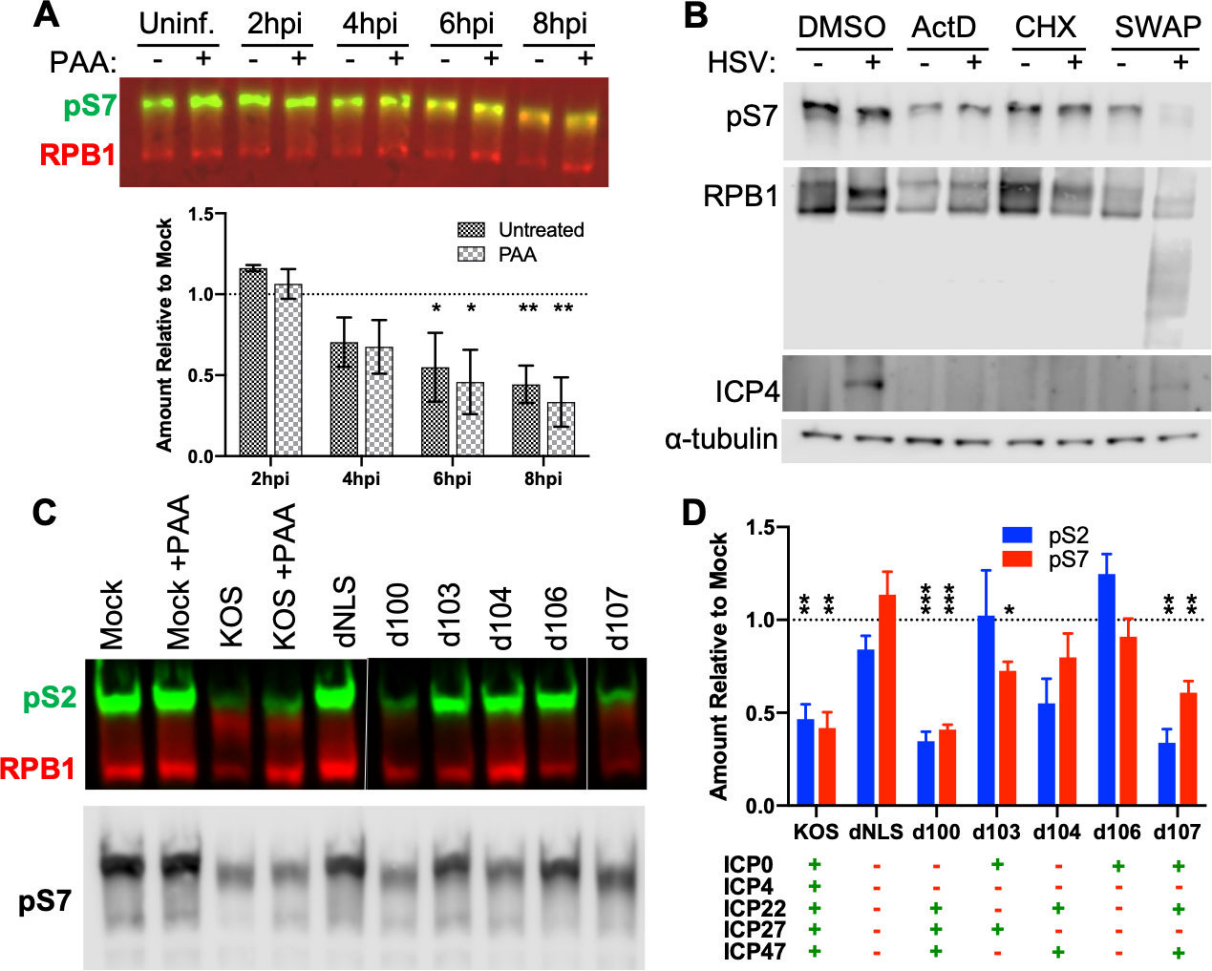
Loss of CTD serine 7 phosphorylation in HSV infection requires immediate-early proteins ICP22 and ICP27. (**A**) Human foreskin fibroblasts (HFF) were infected with HSV-1 17syn+ with and without DNA replication inhibitor phosphonoacetic acid (PAA), and total protein was harvested at the given time points. Levels of CTD serine 7 phosphorylation (pS7) relative to total RPB1 were quantified by Western blotting. Below are means of at least three replicates relative to the corresponding mock-infected sample plotted, with standard deviations. (**B**) HFF cells were infected with indicated HSV mutants at MOI 10 for 1 h, and inoculum replaced with growth media or media containing phosphonoacetic acid (PAA) for 8 h. Total protein levels were analyzed for pS2, pS7, and RPB1. Wild-type (WT) strains F, KOS, and 17syn+ are shown next to corresponding mutants for ICP22 (∆22), ICP4 (∆4), ICP27 (∆27), and ICP0 (∆0). Plotted are means of at least three biological replicates for PAA-treated samples with standard error. (**C**) HFF cells infected with HSV-1 KOS treated with PAA or KOS-derived mutants lacking the combinations of immediate-early genes indicated in the table in the bottom right. Total protein was harvested at 8 h p.i. and pS2/7 levels probed by western blot. White bars indicate the removal of irrelevant samples. (**D**) Quantification of phospho-serine normalized to total RPB1. Means of three replicates normalized to mock-infected samples with PAA with standard error are plotted, and values from KOS are from PAA-treated samples. Statistically significant differences to mock are indicated as *P < 0.05, **P < 0.01, ***P < 0.001.

The loss of pS2 during HSV infection has been linked to the inhibition of CDK9 by the viral immediate-early (IE) protein ICP22 (11). As CDK9 can also phosphorylate Ser7 (3), we investigated the role of ICP22 and other viral gene products in the observed loss of pS7. A time course of infection, with and without the DNA replication inhibitor phosphonoacetic acid (PAA), to prevent viral late gene expression, demonstrated that early viral gene products are sufficient for pS7 loss (Fig. 2A). Viral late genes and/or DNA replication, although not required, could facilitate further CTD remodeling (12, 13, 31). To specifically test immediate-early (IE) genes, a cycloheximide reversal assay was performed. After 4 h, cycloheximide was removed and replaced with actinomycin D for an additional 8 h to allow translation of the accumulated viral IE mRNA but prevent transcription of early and late genes. In these conditions, however, a noticeable decrease in all forms of Pol II due to degradation was observed (Fig. S2A). This is consistent with previous reports using actinomycin D, suggesting that the normal virally induced CTD modifications require ongoing transcription (32).

Of the five viral IE genes, four (ICP0, ICP4, ICP22, and ICP27; all except ICP47) exhibit nuclear localization and can affect transcription; thus, viral mutants lacking each of these four genes were tested for CTD phosphorylation. However, no individual viral IE gene seemed sufficient to account for the full loss of pS7 (Fig. 2B; Fig. S2B). As this suggested the involvement of more than one viral gene product, a panel of viruses lacking the major viral transcription factor ICP4 and combinations of the other IE genes were tested. No loss of pS7 was observed upon infection with a mutant virus whose genome is not trafficked to the nucleus due to the removal of the nuclear localization signal in the UL36 protein (dNLS, Fig. 2C and D). Therefore, viral tegument proteins delivered by the incoming virus particles are not sufficient to induce pS7 loss, as indicated by the retention of pS7 when using cycloheximide alone (Fig. S2A). The strongest reduction (~50%) in pS7 was observed in viruses expressing both ICP22 and ICP27 (KOS and d100, Fig. 2C and D). Viruses lacking either ICP22 (d103) or ICP27 (d104, d107) had an intermediate (~25%) loss of pS7, whereas a virus lacking both proteins (d106) had no reduction. The regions of amino acids 193–256 of ICP22 are sufficient to bind CDK9 (11), and we could confirm this as a major contributor to pS7 loss as smaller mutations, particularly those involving residue Y230, appeared to rescue much of the pS7 (Fig. S2C). Interestingly, a homologous region in other mammalian alphaherpesviruses varicella zoster virus and Cercopithecine herpesvirus 1 exchanged for the HSV-1 sequence resulted in more modest repression in both pS2 and pS7, indicating these regions may not strongly bind or inhibit human CDK9 (Fig. S2C).

The contribution of ICP22 to the loss of both pS2 and pS7 can be attributed to inhibition of CDK9, whereas ICP27’s impact on pS7 is less readily apparent. ICP27 is known to co-precipitate with RPB1 (33, 34) and other transcription factors such as SPT5; the latter interaction facilitated by CDK9 activity (35). ICP27 has also been previously reported to mediate Ser2 phosphorylation in HeLa and RSF (34, 36)—but not Vero (13)—cells. However, as the other immediate-early mRNAs exhibit less cytoplasmic accumulation without ICP27 (37), an effect on ICP22 cannot be ruled out for the impact on pS2. Furthermore, ICP22 can interact with at least four CTD kinases (14) and the relative activity of these may vary in different cell types. These data indicate that although the loss of ICP27 did not alter ICP22-mediated effects on serine 2 phosphorylation in infected human fibroblasts, ICP22 and ICP27 work in a cooperative manner to deplete serine 7 phosphorylation and that they do not require late viral gene expression.

### HSV utilizes multiple proteasome-dependent pathways to regulate RPB1

Early studies have shown that total transcriptional activity in infected cells rapidly shuts down after the peak of viral gene expression around 8 h p.i (38–40), associated with Pol II holoenzyme remodeling (41) and degradation of RPB1 itself at later time points (32). In addition to viral replication compartments, RPB1 localizes to virus-induced chaperone-enriched (VICE) domains (34, 36), which have been proposed to serve as sites of quality control for protein folding and/or complex assembly as they contain numerous chaperones and proteasome components (42, 43). Protease activity and different types of mono- and poly-ubiquitination have been detected in VICE domains, although it is not clear if all proteins trafficked to these compartments are fated for degradation or if these can serve as assembly sites for complicated higher-order protein structures (44).

**Fig 3:**
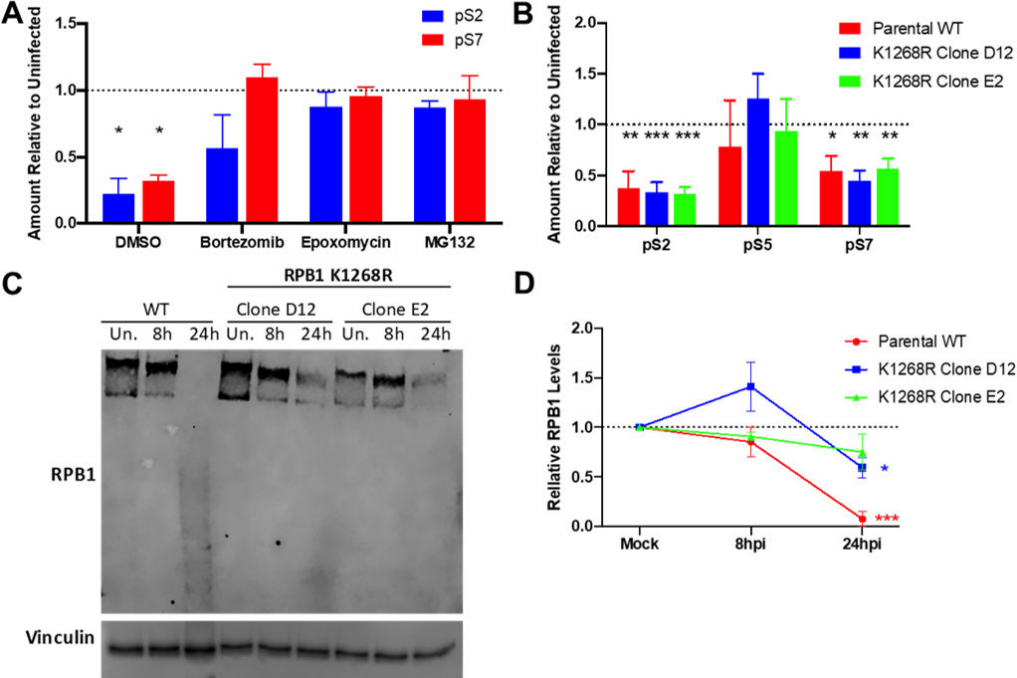
Effects of direct proteasome inhibition and RPB1 polyubiquitination mutants on HSV-1-induced RPB1 remodeling. (**A**) Human foreskin fibroblasts were infected with HSV-1 17syn+ and treated with indicated proteasome inhibitors for 8 h before collection of total protein and quantification of CTD Ser2 and Ser7 phosphorylation. Plotted are means of two replicates with standard deviations. (**B-D**) 293 parental wild-type (WT) and clonally derived RPB1 K1268R mutant cell lines were infected with HSV1(17+)-LoxCheVP26 and total RPB1 or phosphorylation CTD serine residues quantified by western blotting. (**B**) Relative levels of CTD serine phosphorylation at 8 h p.i.; plotted are three individual replicates with lines indicating means and standard error. (**C**) Representative Western blot of RPB1 levels in uninfected (Un.) 293 WT and RPB1 K1268R or cells infected for 8 and 24 h. (**D**) Relative RPB1 levels normalized to Vinculin in infected 293 WT and RPB1 K1268R cells; plotted are means of at least three replicates with standard error. Statistically significant differences in the uninfected are indicated as *P < 0.05, **P < 0.01, ***P < 0.001.

Although the exact roles of VICE domains in regulating transcription are unknown, direct proteasome inhibitors that block VICE domain formation (34, 36) were previously found to prevent the loss of pS2 (34), and we observed this as well for pS7 (Fig. 3A; Fig. S3). Previous work has demonstrated that ICP22 expression is not altered by proteasome inhibition (45). Rice and colleagues have suggested that ICP22-mediated inhibition of pS2 is mechanistically distinct from the degradation of hyperphosphorylated RPB1 (13). To this point, we observed no significant loss of total RPB1 levels by 8 h p.i. or immediately following heat, osmotic, or oxidative stress using five different RPB1 antibodies (Fig. 1; Fig. S4), indicating CTD remodeling as the major effector or degradation of a small percentage of RPB1 molecules (as detectable by western blot), which contain the vast majority of Ser2/7—but not other CTD residue—phosphorylation in the cell.

The most well-studied RPB1 degradation pathway is associated with transcription-coupled repair where elongating Pol II stalls at sites of DNA damage and signals to repair machinery via polyubiquitination of RPB1, causing release of RPB1 from DNA and subsequent degradation. Recent work has demonstrated that this polyubiquitination occurs on a single lysine, K1268, and although other sites of monoubiquitination exist, mutation of this single lysine to arginine completely prevents degradation of RPB1 after UV irradiation (46). Two monoclonal cell lines bearing the RPB1 K1268R mutation exhibited an equal loss of serine 2 and 7 phosphorylation during HSV-1 infection as the parental wild-type (WT) cell line by 8 h p.i. (Fig. 3B; Fig. S5A). As in fibroblasts, no major loss of RPB1 was observed by 8 h p.i. in any of the cell lines. By 24 h p.i., however, RPB1 in WT cells was hardly detectable, migrating almost exclusively as a lower MW smear while RPB1 K1268R mutant cells exhibited a ~50% retention in total RPB1 protein (Fig. 3C and D). Visualization of mCherry fused to the viral late gene VP26 indicated that viral gene expression at 8 and 24 h was comparable across all cell lines, and this effect was not due to global differences in viral gene expression (Fig. S5B). This demonstrates that the RPB1 ubiquitination pathway active during DNA damage is not involved in CTD remodeling during the bulk of viral gene expression but degrades Pol II at later times during virion assembly and genome packaging. It is possible that the 50% reduction of RPB1 in the K1268R cells may not be due to degradation, but standard protein turnover coupled with the virus-induced shutoff of host mRNA translation as RPB1 is reported to have a half-life of 6 h in mammalian cell lines (47). Virus production in these cells was measured to determine if RPB1 degradation facilitates genome packaging. However, the two clones bearing the K1268R mutation gave differential phenotypes as the mutant clone D12 produced viral progeny equal to WT cells, whereas the E2 clone was consistently ~5-fold lower than the other two cell lines (Fig. S5C). Overall, these data indicate that although proteasome-dependent pathways are active in CTD remodeling during lytic HSV infection, they are separate from transcription-coupled repair and precede the bulk degradation of RPB1.

### Differential recruitment and requirements for CTD modifications in sites of herpesviral transcription

**Fig 4:**
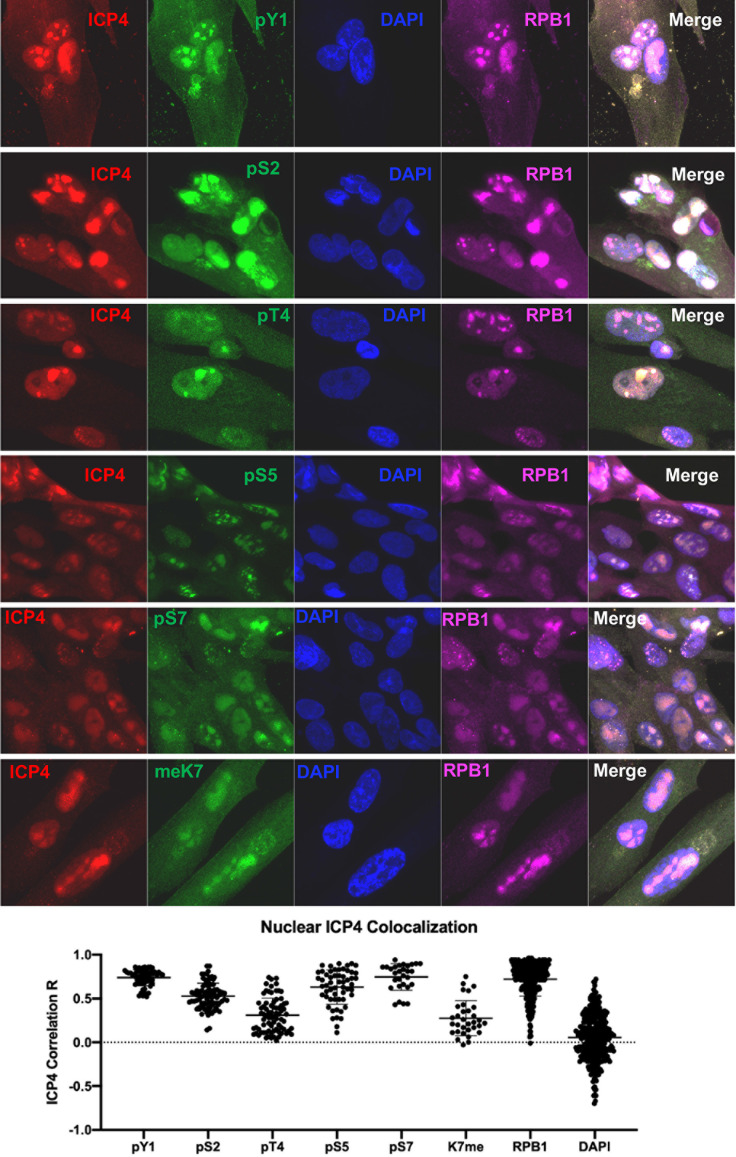
Co-localization of RPB1 CTD modifications with viral replication compartments. Human foreskin fibroblasts were infected with HSV-1 strain 17syn+ for 8 h before fixation. Antibody staining was performed for viral protein ICP4, RPB1, and its respective CTD modifications. p, phosphorylation; me, methylation. Co-localization analysis with ICP4 was performed with ZEN Black microscopy software from Zeiss.

During infection, Pol II is strongly enriched in viral replication compartments (RCs) (9): large compartments predominately assembled by viral DNA and the viral transcription factor ICP4 that recruit numerous factors for transcription, DNA replication, and virion assembly while excluding host proteins such as histones and gene silencing factors (48). We sought to determine which CTD modifications localized to viral DNA by immunofluorescence. Although HSV infection reduced global pS2 and pS7 levels, these modifications were visible in ICP4 foci, whereas other CTD modifications could be localized to varying degrees (Fig. 4). To accurately quantify colocalization within replication compartments, regions of interest were defined as the entire nucleus of ICP4-positive cells and the correlation of RPB1, CTD modifications, and DAPI to ICP4 were measured using Zeiss ZEN microscopy software. Although vastly different sizes of replication compartments could be observed (Fig. 4) and have been previously described (49), we included all infected nuclei to avoid potential bias. As shown in Fig. 4B, RPB1 had high colocalization to ICP4 (R correlation = 0.72). The CTD modifications most closely matching RPB1 were pY1 and pS7 (R values 0.74 and 0.75, respectively). Correlation with pS2 and pS5 was slightly lower (R values of 0.53 and 0.63, respectively), whereas pT4 and K7me exhibited low correlation (R values of 0.31 and 0.28, respectively). The strongest DAPI signals were typically outside of RCs and, while exhibiting the greatest range of values, had no correlation with ICP4 (R = 0.06).

Previous studies have shown that chemical inhibition of CDK9 reduces herpesviral gene expression (50), indicating that HSV still requires some degree of phosphorylation on the CTD and/or other transcription factors. We decided to investigate the requirements of viral genes for CTD serines 2 and 7 using an amanitin-based RPB1 replacement assay. HEK-293T cells were co-transfected with vectors expressing α-amanitin-resistant, HA-tagged RPB1 constructs (51, 52), containing either wild-type (WT) or mutant CTDs and a vector expressing a Pol III-transcribed scrambled control single-guide RNA (sgRNA) that should not be affected at low amanitin concentrations and is used to normalize transfection efficiency. The mutations are non-phospho-accepting alanine substitutions in place of serine 2 or 7 (S2A and S7A, respectively) or the phospho-serine mimic glutamic acid at serine 7 (S7E). After 24 h, α-amanitin was added to degrade endogenous RPB1 for an additional 24 h, followed by infection with HSV for one additional day before harvesting RNA. Western blotting for the plasmid-expressed, HA-tagged RPB1 verified that expressions of the exogenous RPB1 mutants were comparable across conditions at the start of infection (Fig. S6A).

**Fig 5:**
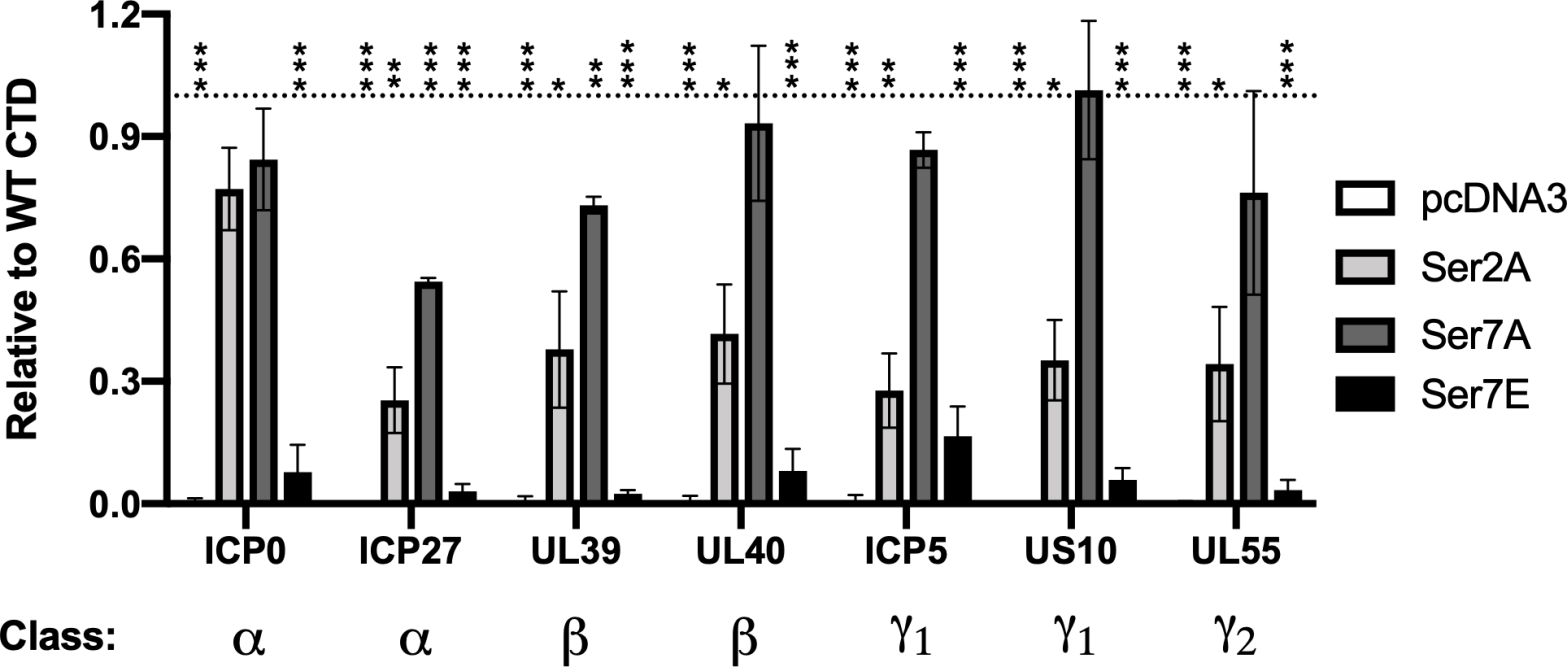
CTD serine 2, but not serine 7, is required for efficient viral gene expression. HEK-293T cells were co-transfected with a construct expressing a sgRNA transcribed by Pol III as a transfection control and plasmids expressing amanitin-resistant RPB1 with wild-type (WT) or indicated mutant CTDs, or the control vector pcDNA3 lacking any polymerase. The next day, α-amanitin was added to degrade endogenous RPB1 for 24 h followed by infection with HSV-1 strain 17+, and viral gene expression was measured by RT-qPCR at 24 hpi. Means of three biological replicates with standard error are plotted. Statistically significant differences to mock are indicated as *P < 0.05, **P < 0.01, ***P < 0.001. S2A: serine 2 to alanine, S7A: serine 7 to alanine, S7E: serine 7 to glutamate.

Across three classes of viral genes, expression with S7A mutations was similar to WT CTD except for slight reductions for ICP27 and UL39, whereas expression with S7E was almost undetectable (Fig. 5). The phosphomimetic S7E mutations ablating viral gene expression, more so than S2A, is likely due to reduced recruitment and/or 3’-end processing as with host genes (52). Viral gene expression in the S2A CTD was reduced compared with WT, especially for late (gamma) genes, but three IE/early genes were similar to the S7A CTD, suggesting that viral immediate-early promoters may be able to recruit S2A mutant CTDs, but there are likely downstream defects in RNA processing and/or termination that prevent these transcripts from becoming mature mRNA and activating the later kinetic gene classes. To interrogate viral protein levels, a virus expressing eYFP-ICP0 as an immediate-early gene marker and gC-mCherry as a late gene marker (53) was used. As shown in Fig. S6B, detectable levels of ICP0 and gC could be visualized in cells with replaced WT CTD at 24 h p.i., albeit at substantially lower levels than amanitin-untreated cells, but not with empty pcDNA3 vector control. As expected, both early and late viral gene expressions were reduced with CTD S2A mutants. In contrast, S7A mutations expressed both classes of viral genes equally well as the WT CTD. Together, these data demonstrate that while replication compartments localize all major CTD modifications, not all modifications are required for viral late gene expression. In the case of CTD serine 7, the amino acid itself appears completely dispensable during lytic infection. Although HSV inhibits Ser2 phosphorylation, at least some level appears required for viral gene expression.

## DISCUSSION

Post-translational modification, particularly phosphorylation, of the RPB1 CTD is at the core of numerous transcriptional responses. Release of CDK9 from the 7SK snRNP and resulting transcription of P-TEFb-dependent genes greatly impacts mammalian cell survival during genotoxic stress (54) and interferon responses during viral infection (55). As phosphorylation of multiple CTD residues (namely Tyr1, Ser2, and Thr4) is involved in the termination of mammalian protein-coding genes, we reasoned that there could be a core dysregulation of the CTD code in response to stress. However, we observed no major changes in the CTD shared across all conditions tested. Both HSV-1 infection and heat stress induced the intermediate-migrating IIi form of RPB1, whereas only HSV showed a consistent loss of pS2 and pS7 with all antibodies tested. Future work should implement mass spectrometry-based assays (2, 56) to reduce bias between different antibodies and quantify the modifications of the intermediately phosphorylated forms of RPB1 in heat stress and HSV infection. Although different challenges to cellular homeostasis can have similar global impacts on gene expression, particularly inhibition of polyadenylation, our data demonstrate that this is not due to a common, global disruption of the RPB1 CTD.

Western blotting of different viral mutants implicates *de novo* expression of both ICP22 and ICP27 in the loss of pS7 in fibroblasts. Although ICP22’s known ability to regulate CDK9 and bind to other CTD kinases can account for this protein’s contribution, ICP27 may boost ICP22 expression and function, or work by other means. Beyond simply supporting the expression of other viral genes, ICP27 binds directly to RPB1 (34), which could disrupt downstream interactions and phosphorylation. Both ICP22 and ICP27 have been implicated in VICE domain formation, and it is possible that RPB1 may interact with proteins in these domains, which limits its ability to be phosphorylated. Although VICE domains contain numerous components involved in protein misfolding and degradation, we favor the view that they facilitate RPB1 remodeling and trafficking over proteolysis as we do not observe a major decrease in RPB1 levels until well after the decrease in pS2/7. Previous studies using mass spectrometry to identify proteins interacting with ICP22 (14, 57) and ICP27 (10, 58) did not detect any of the major CTD phosphatases; therefore, we propose that these two proteins do not directly recruit phosphatases to reduce levels of pS2/7.

Our data support previous observations that ongoing viral transcription is required for CTD remodeling, as even relatively weak expression of immediate early genes can trigger RPB1 degradation in cycloheximide/actinomycin D reversal assays. This degradation pathway is likely separate from the one during DNA damage as it occurs when transcription is chemically inhibited. We observed that degradation of RPB1 mediated by polyubiquitination of K1268 is not involved in the loss of pS2/7 but moderately stabilizes RPB1 24 h p.i. This suggests that there are multiple degradation pathways triggered by HSV at different stages of infection and reside in a balance with ongoing viral transcription or trafficking between VICE domains and replication compartments (RCs). Separate pathways for degrading promoter-proximal and productively elongating polymerases in cells have been proposed (59). Degrading Pol II on viral DNA may resolve conflicts resulting from polymerases transcribing overlapping genes in opposite orientations or preserve genomes in settings of abortive infection by halting the production of viral antigens or innate immune triggers like double-stranded RNA and even help maintain latency in the presence of leaky transcription. It would be of interest to experimentally determine ubiquitination sites at early and late time points of HSV infection by mass spectrometry to better understand how the proteasomal machinery is involved with viral transcription cycles and shuttling of RPB1 between the various nuclear compartments.

The interaction of ICP22 and CDK9 causing reduced transcriptional elongation rates of cellular mRNA has been proposed to benefit HSV as a means of countering host responses during infection. However, chemical inhibition of CDK9 also reduces viral gene expression, and our data with S2A mutants recapitulate this phenotype, indicating a beneficial role for at least intermediate pS2 levels to viral transcription. Contrary to this, polymerases with S7A mutations expressed viral proteins comparable with the WT CTD, demonstrating that the entire CTD code is not required for herpesviral gene expression despite all tested modifications being localized to viral replication compartments. The relative quantities of each CTD modification transcribing viral genes require immunoprecipitation and sequencing-based assays and will be the focus of future work. We cannot rule out the possibility that Ser7 regulation may be more impactful during reactivation from latency over lytic infection or during inflammatory responses. Indeed, inhibition of another Ser7 kinase, CDK7, has been shown to reduce cytokine release and inflammation (60, 61), but more work is required to determine contributions from Ser7 vs. other CDK7 targets, especially CTD Ser5.

Currently, the best-described role of pS7 is recruitment of the integrator complex to snRNA genes (62) for 3’-end formation of Pol II-derived snRNA (63). Disrupting snRNA formation by itself is unlikely to influence lytic infection as the rapid redirection of Pol II activity to viral genomes and transcriptional shutdown within the span of 8–12 h would not be expected to alter the abundant pool of mature splicing factors, and most viral genes are unspliced late in infection when defects in mRNA 3’-end formation are the greatest. Furthermore, an increase in U4 snRNA transcription due to VP16 was previously observed, whereas that of U2 was unaffected (64). Integrator also has direct roles in regulating protein-coding genes, and knockdown of its catalytic subunit recapitulates termination defects on a subset of host mRNAs disrupted during osmotic stress (65), although our previous work implicates ICP27-CPSF interactions as the primary determinant of pre-mRNA termination failure during HSV infection (10).

The reduction of pS7 could also result in more subtle, global consequences on host gene expression as pS7 has been proposed to suppress cryptic transcription (66) as well as stimulate Ser2 phosphorylation by CDK9 and CDK12 (67, 68). As viral genes are much shorter and in closer proximity to each other relative to those of the host, we propose that limiting CTD phosphorylation could dampen transcription after termination sites into downstream genes by slowing Pol II elongation or helping eject it from chromatin. Furthermore, partially phosphorylated CTDs may be more rapidly recycled to initiation sites, and this could support the frequent, transient exploration of DNA, which localizes Pol II to replication compartments (9). Our future efforts will involve mapping the genomic locations of pS2/7 during infection by ChIP/mNET-seq to better understand the impacts of herpesviral-induced CTD remodeling.

## MATERIALS AND METHODS

### Cell lines and viruses

Human foreskin fibroblasts were purchased from ECACC and ATCC. BHK-21 cells were purchased from ATCC. LUHMES cells were gifts of David Bloom, University of Florida. Flp-In T-Rex HEK-293 RPB1 K1268R clones D12 and E2 as well as the corresponding parental cell line (46) were gifts from Jesper Svjestrup, Oxford University. Vero cells were provided by Beate Sodeik, Hannover Medical School; Vero derivatives E5 (69) and F06 (70) were gifts from Neal A. DeLuca, University of Pittsburg; Vero 2–2 (71) from Rozanne M. Sandri-Goldin, UC Irvine; and ICP22-expressing Vero V22 cells (72) provided by Stephen Rice, University of Minnesota. RSC and RSC-HAUL36 (73) were provided by Peter O’Hare, Imperial College London. HEK-293T cells were provided by Manfred Lutz, University of Würzburg. All cells, except for LUHMES described below, were cultured in Dulbecco’s Modified Eagle Medium (DMEM), high glucose, pyruvate (ThermoFisher #41966052) supplemented with 1× MEM Non-Essential Amino Acids (ThermoFisher #11140050), 1 mM additional sodium pyruvate (ThermoFisher #11360070), 10% (vol/vol) Fetal Bovine Serum (FBS, Biochrom #S 0115), 200 IU/mL, penicillin, and 200 µg/mL streptomycin. All cells were incubated at 37°C in a 5% (vol/v) CO_2_-enriched incubator.

LUHMES cells were cultured in flasks sequentially pre-treated with poly-L-ornithine (50 µg/mL) overnight at room temperature and 1 mg/mL fibronectin overnight at 37°C. They were maintained in proliferation media (DMEM:F12 with L-glutamine, Sigma D8437) containing 1% N2 supplement CTS (Life Technologies #A1370701), 1× Penicillin-Streptomycin-Glutamine solution (ThermoFisher Sci #10378016), and 40 ng/mL recombinant human FGF-basic (Fibroblast Growth Factor, Peprotech #100–18B) and passaged by 75% confluency. Differentiations were induced in the same media but FGF was replaced with 1 µg/mL tetracycline hydrochloride (Sigma #T7660), 1mM N6,2´-O-Dibutyryladenosine 3´,5´-cyclic monophosphate sodium (Sigma #D0627), and 2 ng/mL recombinant human Glial cell-derived neurotrophic factor (GDNF, R&D Systems #212-GD-010). Infections were performed at least 5 days post-differentiation with media changes every 2 days, beginning with 100 K cells initially seeded in 6-well dishes followed by 2 days of proliferation before differentiation.

KOS strain ICP4 mutant n12 (69) provided by Neal DeLuca and KOS 1.1 ICP27 mutant 27-lacZ (71) provided by Rozanne M. Sandri-Goldin were propagated on E5 cells and Vero2-2 cells, respectively. KOS BAC-derived VP1-2∆NLS (74) was cultured as previously described in RSC cells. Wild-type strains 17syn + and F, along with BAC-derived strains KOS and HSV1(17+)-LoxCheVP26 (75), were propagated on BHK-21 cells as well as strain 17 ICP0 mutant dl1403 (76) from Roger Everett, strain F ICP22 mutant ∆325 (77) from Bernard Roizman, and the KOS eYFP-ICP0/gC-mCherry BAC-derived virus (53), which was provided by Colin Crump, University of Cambridge. KOS mutants d100, d103, d104, d106, and d107 (78), generously provided by Neal DeLuca, were grown and titered on F06 cells. A lack of plaque formation on non-complementing Vero cells was monitored as controls for recombination in viruses lacking essential genes.

The BAC-derived ICP22 mutants are based on the pHSV1(17+)Lox BAC (75) and were cloned in two steps. First, the US1 ORF was destroyed by removing the bases between positions 153340 and 154613 of the BAC using *en passant* mutagenesis (79) via a series of intermediate vectors. For this, the V5-tagged HSV-1 US1 was cloned from pHSV1(17+)Lox into a modified doxycycline-inducible lentivirus system designated TH3 (37). Chimeras replacing the HSV-1 US1 core region (aa187-259) with that of VZV (53-125) and CeHV-1 (205-276) were generated by In-fusion HD cloning (Takara). The generated US1 derivatives were transferred into a BAC targeting vector based on pEP-Kan-S at the SgrDI site. Herpes B DNA was a kind gift from Prof. S. Pöhlmann. Although the Herpes B virus strain is not known, the sequence of the regions completely overlaps with strain E2490 (accession KY628984.1). The VZV DNA isolated from Zoster patient material was a gift from Prof. Dr. M. Goebeler and the regions match the sequence of accession MH709377.1. Virus stocks were generated through transfection of the purified BAC DNA into BHK cells then further amplified and titered on V22 cells.

For infection experiments, 293 cell lines were seeded onto dishes that were previously coated with poly-lysine for 1 h. All infections were performed with an MOI 10. Virus was diluted in a differentiation medium for LUHMES and given to cells after differentiation, whereas all other cell lines were infected in serum-free DMEM the day after seeding. After 1-h adsorption at 37°C, the inoculum was replaced with culturing medium or medium containing concentrations of compounds described below.

### Stress and drug treatments

Heat stress was initiated by adding pre-warmed 44°C medium to cells and culturing for 2 h at 44°C. Salt stress and oxidative stress were initiated by adding KCl or sodium arsenite to concentrations of 80 mM and 0.5 mM, respectively, for 1 h. Mock cells were harvested at time point 0 and infected cells at 8 h p.i.

Phosphonoacetic acid (PAA, Sigma #284270) was used at a concentration of 300 µg/mL when indicated. Bortezomib (Selleckchem #S1013) was used at a concentration of 5 µM; epoxomycin (Cayman Chemical #10007806) and MG132 (Sigma #M7449) both at 10 µM, with equal volumes of DMSO vehicle were used as control. Actinomycin D was used at 5 µg/mL and cycloheximide at 100 µg/mL for the times indicated in the text. Uninfected cells for drug treatments were harvested at the final timepoints as infected samples.

### Western blotting

A full description of antibodies is found in Table S1. Total protein samples were harvested by lysing cells directly in Laemmli buffer at given time points. Samples for RPB1 blotting were resolved on 6% tris-glycine SDS PAGE gels with a 4% stacking gel, both 37.5:1 acrylamide/bisacrylamide, and transferred overnight in tris-glycine buffer containing 20% (vol/vol) methanol to 0.45 µm nitrocellulose membranes. Membranes were rinsed with deionized water and blocked in tris-buffered saline with 0.1% (vol/vol) Tween 20 (TBST) with 5% (wt/vol) milk for 1 h before overnight binding with primary antibodies diluted in blocking buffer. Samples were washed in TBST, blocked for one additional hour, and secondary antibodies were allowed to bind for 1 h before final TBST washes and visualization on a LI-COR Odyssey Fc imaging system. Deviations from this procedure are indicated in the relevant figure legends.

Band densitometry was performed using Image Studio Light (LI-COR). Total RPB1 levels for each sample were normalized to the vinculin signal on the same membrane. Signals for each CTD modification were normalized to total RPB1 levels on the same membrane. The relative ratios are compared with mock samples on the same membrane, which was set to 100%. Statistical significance was determined using the Holm-Sidak method in GraphPad Prism 8.

### Immunofluorescence

3 × 10^4^ HFF cells were seeded onto eight well glass chamber slides (Ibidi #80841). The following day, cells were infected at an MOI of 10 and fixed at 8 h p.i. in 4% formaldehyde in PBS for 15 minutes. After washing in PBS, cells were incubated in permeabilization buffer (10% [vol/vol] FBS, 0.5% [vol/vol] Triton X-100, 250 mM glycine, 1× PBS) for 10 minutes, then blocked for 1 h in blocking buffer (10% [vol/vol] FBS, 250 mM glycine, 1× PBS). Primary antibodies were incubated overnight at 4°C in 10% (vol/vol) FBS and 1× TBS. The secondary antibodies were incubated in 10% FBS in 1× TBS for 1 h at room temperature with 1 µg/mL 4',6-diamidino-2-phenylindole (DAPI). A full description of antibodies is found in Table S1. Coverslips were washed in water before mounting them in a medium containing Mowiol 4–88 and 2.5% (wt/vol) 1,4-diazabicyclo[2.2.2]octane (DABCO). All steps before antibody binding were followed by three 5-minute washes in PBS, whereas TBS was used after antibody binding. Samples were imaged on a Zeiss LSM 780 where slices of 0.5 µm were taken and intensities of each slice summed in Fiji (80) to produce the 2D images provided. Colocalization with ICP4 was calculated in ZEN Black (Zeiss) using default parameters by marking entire ICP4-positive nuclei as regions of interest. Live-cell imaging of fluorescent virus infections was performed on a Leica DMi8.

### RPB1 replacement assay and associated plasmids

5 × 10^4^ HEK-293T cells were seeded in coated 48-well plates as described above. The next day, cells were transfected with 15 ng of pLV-Azurite (gift of Pantelis Tsoulfas, Addgene #36086) and 250 ng of vectors expressing HA-tagged, amanitin-resistant RPB1 constructs with the mutant CTDs or pcDNA3 as a negative control via polyethylenimine (PEI). The WT and S2A mutants are described in (51), and the S7A and S7E mutants in (52). All RPB1 constructs were generous gifts of Dirk Eick, LMU Munich. After 24 h, media was exchanged for fresh growth medium with 2.5 µg/mL α-amanitin for an additional 24 h. Cells were infected with eYFP-ICP0/gC-mCherry HSV at MOI 10 as described above in a serum-free medium for 1 h. This inoculum was removed and amanitin-containing growth medium was reapplied until imaging 24 h p.i.

For qPCR analysis, the assay was repeated but scaled up to 24-well plates, this time using 50 ng of the vector pU6.scr_sgRNA in place of Azurite, which expresses a scrambled control sgRNA based on Addgene #62285 from the RNA Pol III promoter U6. This vector was created by PCR amplifying the U6 promoter from pLKO.1 and cloning it into an XhoI/NdeI fragment of pQE-30 containing ampicillin resistance and an origin of replication. This pU6 vector was then digested with AgeI and EcoRI and a fragment containing the sgRNA from annealing and extending oligomers listed in Table S2 was inserted.

### RT-qPCR

Cells were harvested in TRI Reagent (Sigma), and RNA was isolated according to the manufacturer’s instructions. RNA was DNase treated with the TURBO DNA-free Kit (ThermoFisher #AM1907) and converted to cDNA with random hexamers using SuperScript III First-Strand Synthesis System (ThermoFisher #18080051), including RNase H treatment. cDNAs were then PCR amplified with SYBR green qPCR Master Mix (Bimake #B21202) using the recommended three-step protocol and 1 µM concentrations of the primer pairs listed in Table S2 on a Roche LighCycler 96. Primer efficiencies for each set were validated to be near 100% (Fig. S7). Cq values for each gene were calibrated to the scr_sgRNA for that sample, and relative expression between samples was calculated using the ΔΔCq method. Statistical significance was determined using the Holm-Sidak method in GraphPad Prism 8.

## Data Availability

Data supporting the findings of this study are available from the corresponding author upon reasonable request.

## SUPPLEMENTAL MATERIAL

Supplemental figures jvi.01178-24-s0001.pdf Figures S1 to S7.

Table S1 jvi.01178-24-s0002.xlsx Antibodies used in this study.

Table S2 jvi.01178-24-s0003.xlsx Primers used in this study.

ASM does not own the copyrights to Supplemental Material that may be linked to, or accessed through, an article. The authors have granted ASM a non-exclusive, world-wide license to publish the Supplemental Material files. Please contact the corresponding author directly for reuse.
