# Supplementary material for: Herpes simplex virus 1 inhibits phosphorylation of RNA polymerase II CTD serine-7
Source: J Virol. 2024 Sep 24;98(10):e01178-24. doi: 10.1128/jvi.01178-24 (PMC11494995; doi:10.1128/jvi.01178-24)
Supplement: Supplemental figures — Figures S1 to S7. [file jvi.01178-24-s0001.pdf]

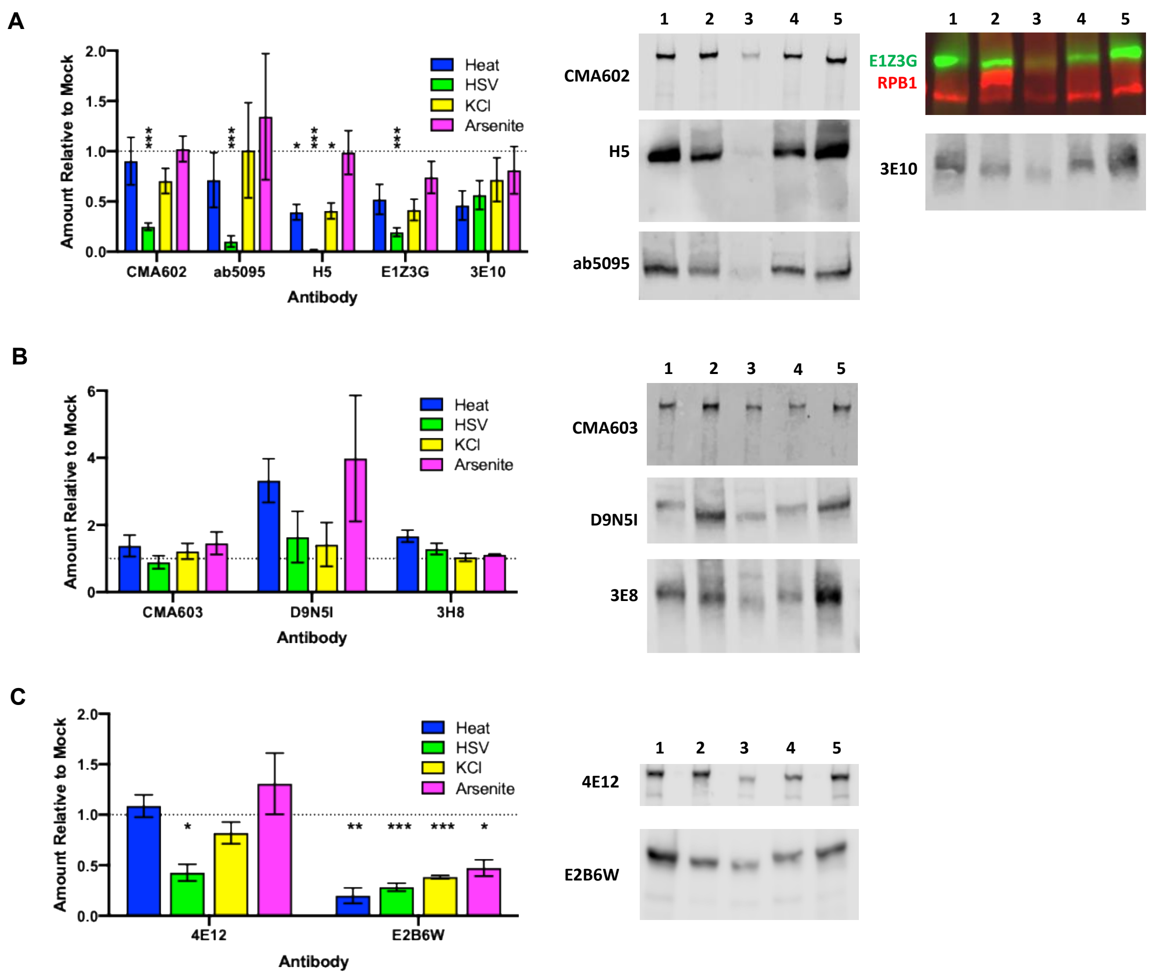

Figure S1. Measurement of RPB1 CTD serine phosphorylation after exposure to stress using different antibodies.

Human foreskin fibroblasts were subjected to mock treatment (lane 1), heat stress (lane 2; 44°C, 2h), HSV infection (lane 3; strain 17syn+, MOI 10, 8h p.i.), osmotic (lane 4; 80mM KCl, 1h), and arsenite (lane 5; 0.5mM $NaAsO_2$, 1h) treatment and total protein harvested at the end of the stress period. Quantification of CTD serine 2 (**A**), serine 5 (**B**), and serine 7 (**C**) phosphorylation was performed using the listed antibodies via Western blotting. Phospho-serine levels were normalized to total RPB1 levels and the relative ratio compared to mock-treated samples (lane 1) on the same membrane. Plotted are the means of three biological replicates with standard error, with representative Western blots on the right. Statistically significant differences to mock are indicated as * $p < 0.05$, ** $p < 0.01$, *** $p < 0.001$.

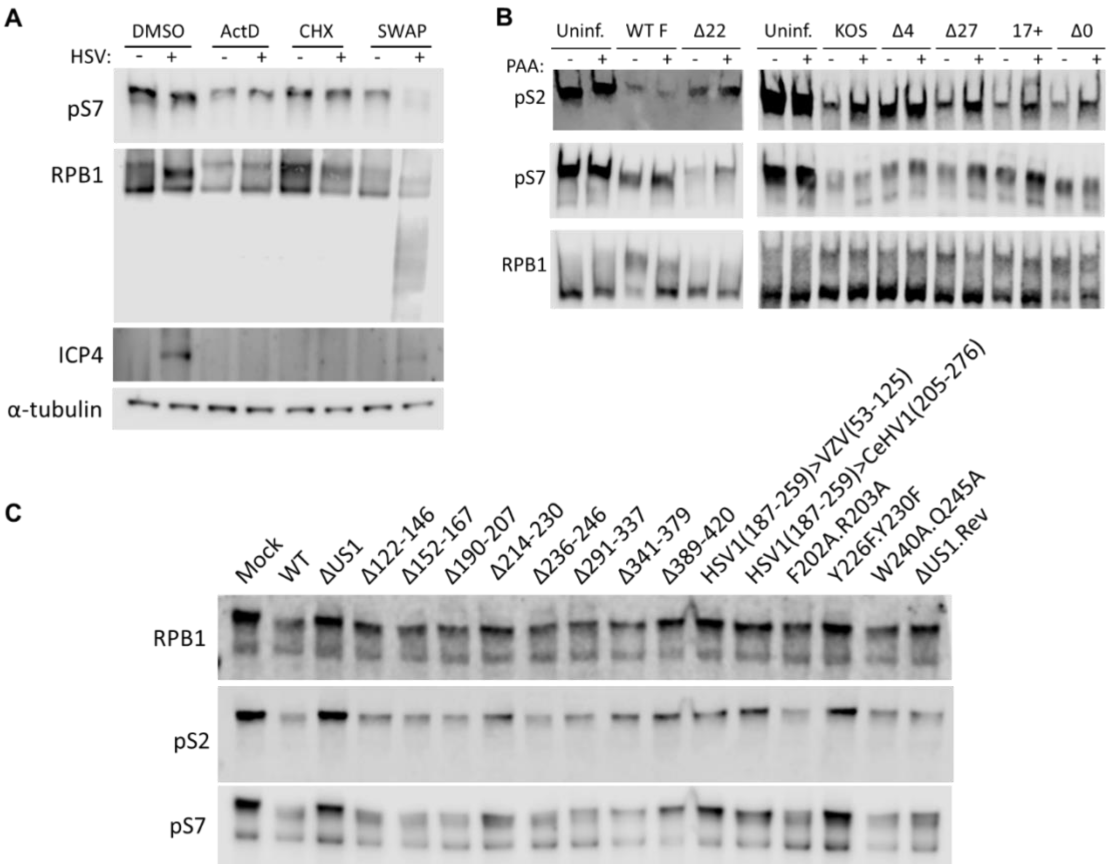


```
VZV        weihparlvedinrvflciaqssgrvtrdsrrlrricldfylmgrtrqrptlacweellqlqptqt-qclratl
HSV-1      wtpdlgymrqcinqlfrvlrvar-dphgsanrlrhlirdcylmgycrarlaprtwcrllqvsggtwgmhlrnti
CeHV-1     weldlpymrrsinqmfrllrrsa-dphgaanrlrrlirdsylmgytrrrlepgmwshmlqvgggr-afrlrnvi
           *  .   : . **::*  :  :      :.***::  * ****  * *     *  .:**:         ** .:
```

Figure S2. CTD serine 7 phosphorylation in cycloheximide reversal and infection with HSV immediate-early gene mutants.

(**A**) Human foreskin fibroblast (HFF) cells infected with HSV-1 17syn+ and treated with DMSO, actinomycin D (ActD), cycloheximide (CHX), or cycloheximide reversal (SWAP; 4h CHX followed by 8h ActD) and total protein analysed at 12h p.i. for levels of CTD Ser7 phosphorylation (pS7) and each indicated protein. Samples for total RPB1 were resolved on 3-8% Tris-Acetate gels to better capture lower molecular weight degradation products. (**B**) HFF cells infected with indicated HSV mutants at MOI 10 for 1h, and inoculum replaced with growth media or media containing phosphonoacetic acid (PAA) for 8h. Total protein levels analyzed for pS2, pS7, and RPB1. Wild-type (WT) strains F, KOS, and 17syn+ are shown next to corresponding mutants for ICP22 (Δ22), ICP4 (Δ4), ICP27 (Δ27), and ICP0 (Δ0). (**C**) ICP22 mutant viruses were created and used to infect HFF cells as described above. Shown are the sequence alignments for the homologous regions in VZV and CeHV-1.

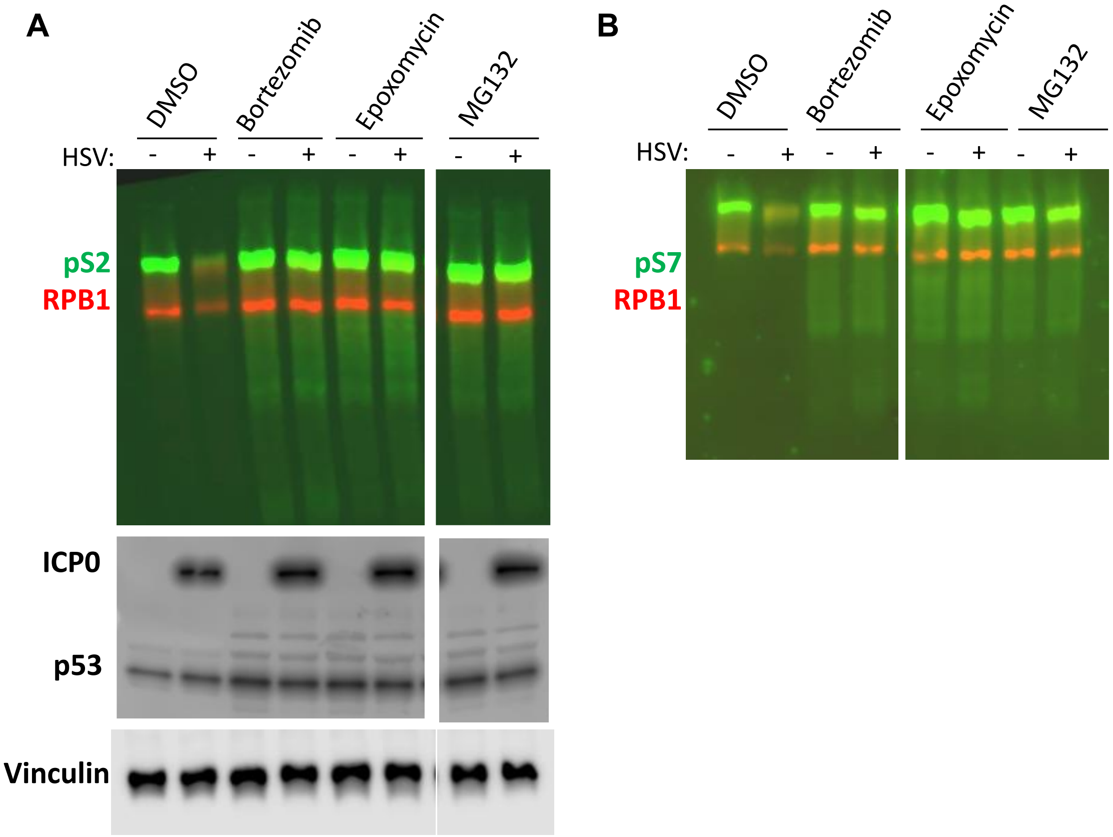

Figure S3. Proteasome inhibition blocks the loss of CTD serine 2 and serine 7 phosphorylation during HSV-1 infection.

Human foreskin fibroblasts were infected with HSV-1 17syn+ at an MOI of 10 and treated with indicated proteasome inhibitors or DMSO vehicle control. After 8 hours of treatment, total protein was collected and analysed by Western blotting. (**A**) Expression of RPB1 (red), phosphorylated CTD Ser2 (pS2, green), vinculin, p53 as a control for inhibitor efficacy, and HSV protein ICP0. (**B**) Expression of RPB1 (red) and phosphorylated CTD Ser7 (pS7, green) by Western blotting. White lines are the result of cropping of irrelevant samples from the gel.

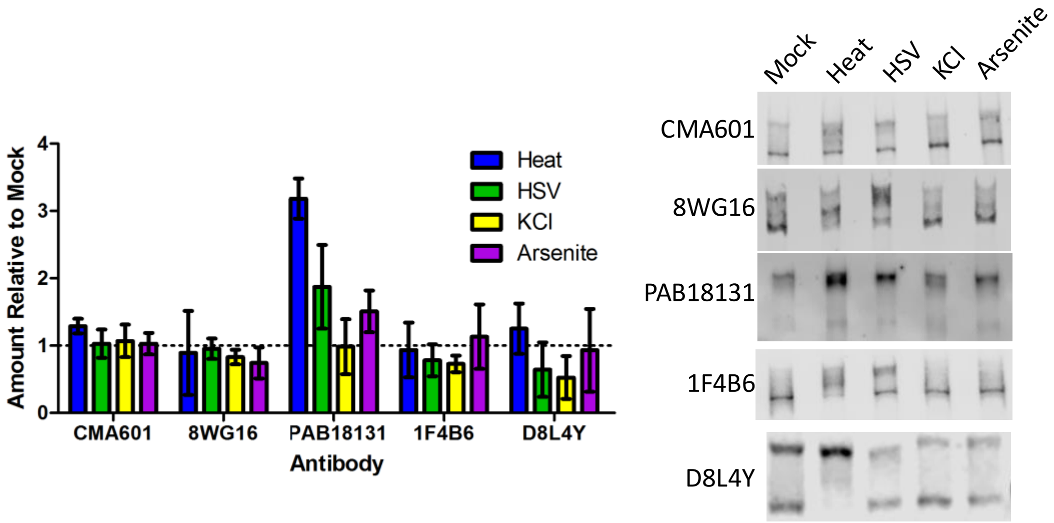

Figure S4. Quantification of RPB1 levels after stress using different antibodies.

Human foreskin fibroblasts were subjected to heat (44°C, 2h), HSV infection (strain 17syn+, MOI 10, 8h p.i.), high salt (80mM KCl, 1h), and arsenite (0.5mM $NaAsO_2$, 1h) treatment and total protein harvested at the end of the stress period. Quantification of RPB1 was performed using the listed antibodies via Western blotting and normalizing to Vinculin levels on the same membrane. Plotted are the means of three biological replicates with standard deviations with representative Western blots to the right. Statistically significant differences to mock are indicated as ** $p < 0.01$.

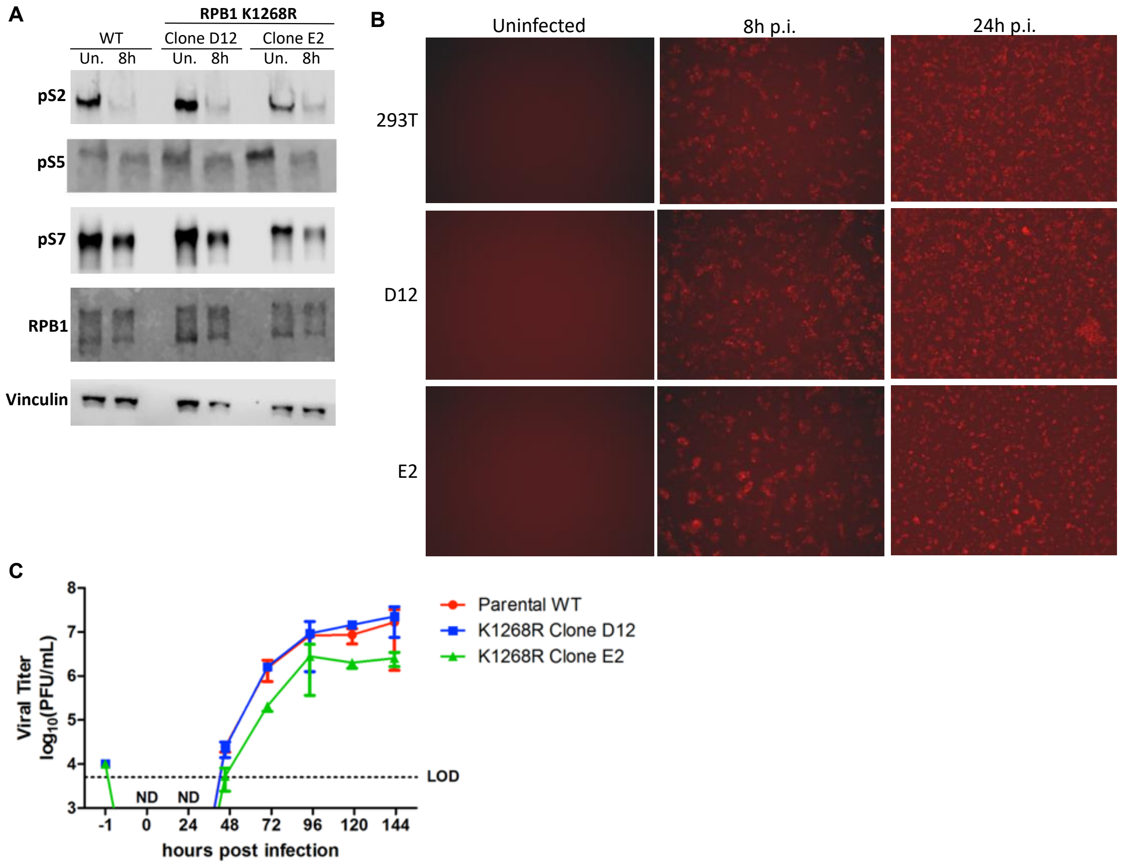

Figure S5. RPB1 serine CTD phosphorylation and viral replication in HSV-1-infected RPB1 K1268R cells.

Clonal cell lines D12 and E2 bearing the RPB1 K1268R mutation and parental wild-type (WT) 293T cells were infected with HSV1(17+)-LoxCheVP26 expressing the VP26 viral late gene fused to mCherry and harvested at 8h p.i. (**A**) Representative Western blots of CTD serine phosphorylations from data graphed in Fig. 3. (**B**) Viral gene expression at analysed timepoints is comparable across cell lines as viewed by live-cell imaging of mCherry-VP26 fusion protein. (**C**) Time course of HSV strain 17+ production from an initial infection of MOI 0.01. Plotted are the means of two replicates with standard deviations. PFU, plaque-forming units; ND, no plaques detected for this timepoint; LOD, statistical limit of detection defined by 10 plaques at lowest dilution.

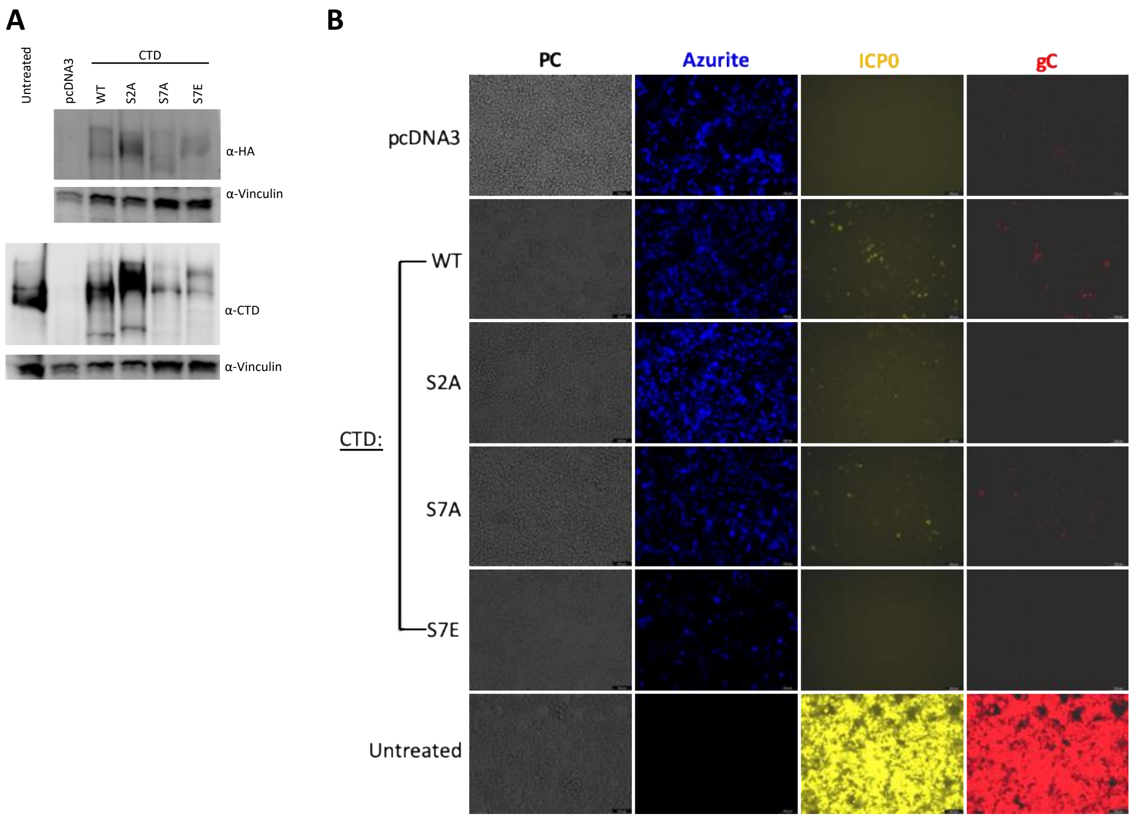

Figure S6. Comparison of RPB1 and fluorescent viral protein levels in the amanitin-based replacement assay.

(**A**) Cells treated for the RPB1 replacement assay described in Fig. 5 were harvested at the time of infection and probed for total CTD levels, the HA tag from the *trans*-expressed RPB1 constructs, and vinculin as a housekeeping gene. (**B**) The assay was repeated but using a plasmid expressing Azurite blue fluorescent protein in lieu of the sgRNA, and infected with an eYFP-ICP0-/gC-mCherry-expressing HSV. Live-cell imaging was performed at 24h p.i. All images were acquired with the same settings, with infection of untransfected, amanitin-untreated cells included as a reference for the relative level of viral gene expression. PC: phase contrast, S2A: serine 2 to alanine, S7A: serine 7 to alanine, S7E: serine 7 to glutamate. Scale bars indicate 100μm.

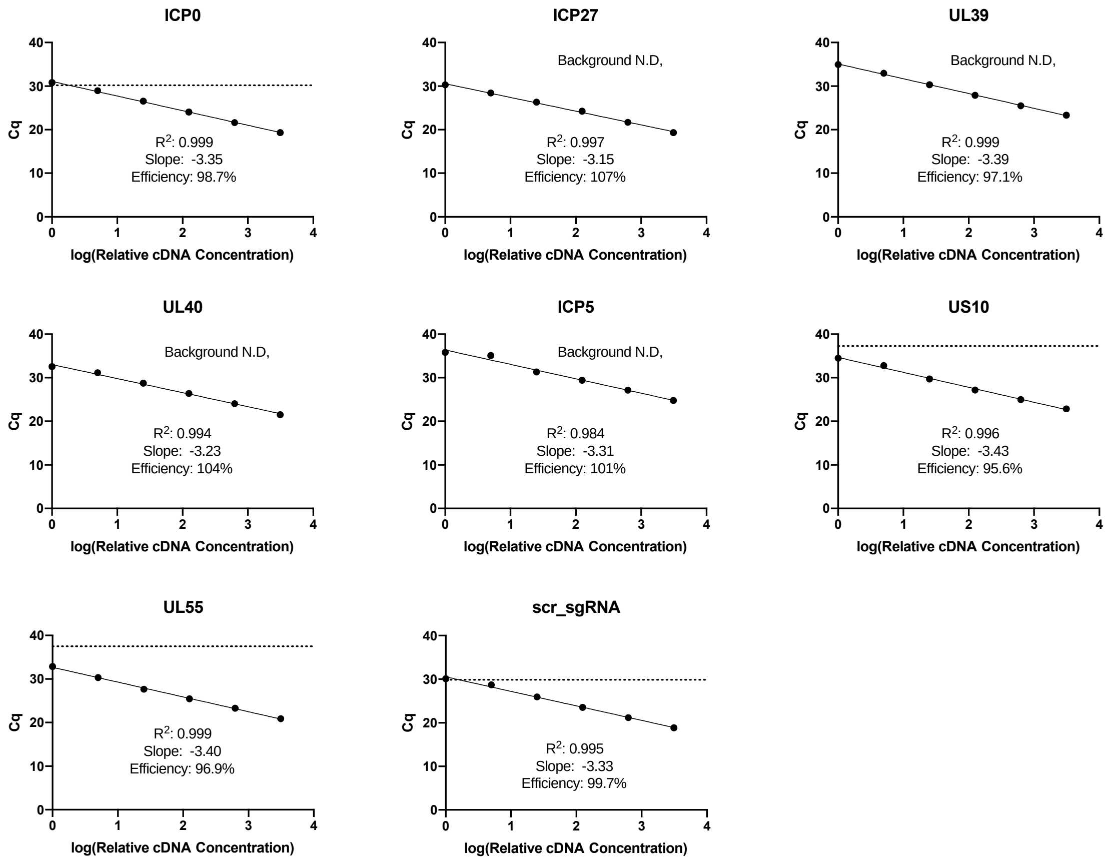

Figure S7. qPCR primer efficiency curves.

cDNA from HSV-infected or sgRNA-transfected cells was serially diluted and tested for amplification efficiency. Dotted lines indicated the values from no template or uninfected/untransfected controls, whichever was lowest. N.D., not detected within 40 cycles.
